# A Guide to the Instruments and Appliances Required in Various Operations

**Published:** 1889-06

**Authors:** 


*A Guide to the Instruments and Appliances required in various Operations.* By A. MAYO ROBSON, F.R.C.S. Pp. 57. London: J. & A. CHURCHILL. 1889.

Mr. Robson has prepared this handy little work as a guide to house-surgeons and dressers who are supposed to get ready the instruments for surgical operations. The book admirably fulfils its intentions. We have thought that, if the operations were arranged alphabetically, reference would be more easy and rapid; but an excellent index leaves little to complain of in this respect.

Surgeons, like other learned men, differ in their method of instructing their pupils. We, for instance, consider that one good means of teaching a dresser or house-surgeon is to think out or read up the details of an operation for himself, and get out the necessary instruments for each step. A book like this goes some way towards making the dresser's duties easy and their discharge accurate; but we doubt if it makes them to the fullest extent educational.

